# Bis(2,2′-bipyridyl-κ^2^ *N*,*N*′)bis­(dicyanamido-κ*N*)manganese(II)

**DOI:** 10.1107/S1600536812014183

**Published:** 2012-04-13

**Authors:** Haixia Wang, Shaohong Wang, Yuehe Lang

**Affiliations:** a Department of Chemistry and Environmental Science, Henan Normal University, Xinxiang 453007, People’s Republic of China

## Abstract

In title complex, [Mn(C_2_N_3_)_2_(C_10_H_8_N_2_)_2_], the Mn^II^ ion is coordinated in a slightly distorted octa­hedral geometry by six N atoms. Four of the N atoms are from two chelating bipyridine ligands and two are from a pair of *cis*-coordinated dicyanamide ligands. The dihedral angle formed by the mean planes of the bipyridine rings is 85.93 (14)°. The central N atom of one of the dicyanamide ligands was refined as disordered over two sites with equal occupancies.

## Related literature

For related structures, see: Lopes *et al.* (2011 ▶); Knight *et al.* (2010 ▶); McCann *et al.* (1998 ▶); Lumme & Lindell (1988 ▶); Li *et al.* (2002 ▶).
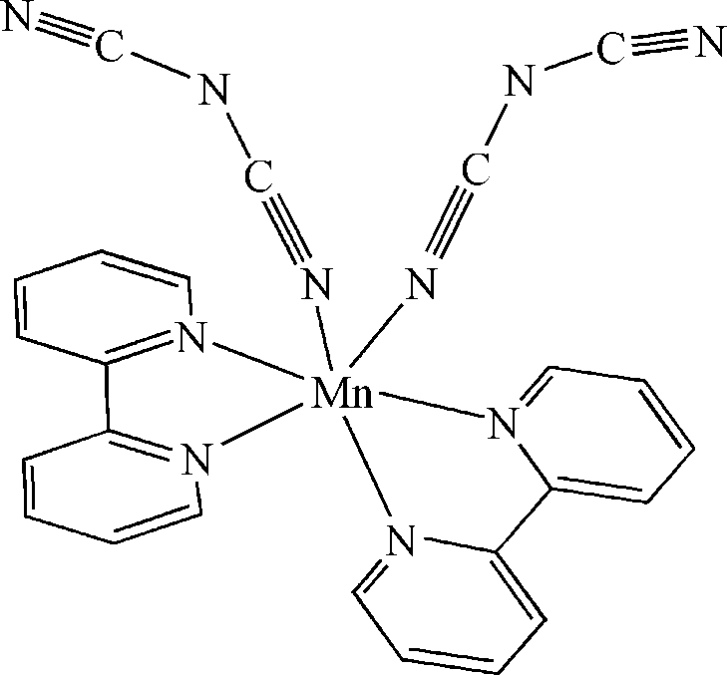


## Experimental

### 

#### Crystal data

[Mn(C_2_N_3_)_2_(C_10_H_8_N_2_)_2_]
*M* *_r_* = 499.41
Monoclinic, 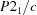

*a* = 9.232 (3) Å
*b* = 16.144 (6) Å
*c* = 16.670 (6) Å
β = 104.439 (6)°
*V* = 2406.0 (15) Å^3^
*Z* = 4
Mo *K*α radiation
μ = 0.58 mm^−1^
*T* = 293 K
0.31 × 0.29 × 0.24 mm

#### Data collection

Bruker APEXII diffractometer
Absorption correction: multi-scan (*SADABS*; Sheldrick, 1996 ▶) *T*_min_ = 0.840, *T*_max_ = 0.873
11521 measured reflections
4220 independent reflections
2653 reflections with *I* > 2σ(*I*)
*R*_int_ = 0.040

#### Refinement

*R*[*F* ^2^ > 2σ(*F* ^2^)] = 0.056
*wR*(*F* ^2^) = 0.178
*S* = 1.03
4220 reflections
325 parameters
H-atom parameters constrained
Δρ_max_ = 0.91 e Å^−3^
Δρ_min_ = −0.79 e Å^−3^

### 

Data collection: *APEX2* (Bruker, 2004 ▶); cell refinement: *SAINT* (Bruker, 2001 ▶); data reduction: *SAINT*; program(s) used to solve structure: *SHELXS97* (Sheldrick, 2008 ▶); program(s) used to refine structure: *SHELXL97* (Sheldrick, 2008 ▶); molecular graphics: *PLATON* (Spek, 2009 ▶); software used to prepare material for publication: *SHELXTL* (Sheldrick, 2008 ▶).

## Supplementary Material

Crystal structure: contains datablock(s) I, global. DOI: 10.1107/S1600536812014183/lh5441sup1.cif

Structure factors: contains datablock(s) I. DOI: 10.1107/S1600536812014183/lh5441Isup2.hkl

Additional supplementary materials:  crystallographic information; 3D view; checkCIF report

## Figures and Tables

**Fig. 1.**
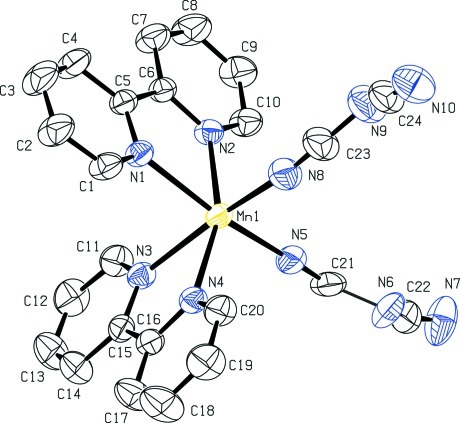
The molecular structure of the title complex with 30% displacement ellipsoids. The disorder is not shown.

## supplementary crystallographic information

### Comment

Due to the excellent chelating abilities to almost all the transition metal ions, 2,2'-bipyridine and its analogs have been widely introduced into coordination chemistry to construct homo- or heterometallic complexes with various structures. Here, we present the crystal structure of the title complex.

The molecular structure of the title complex is shown in figure 1. The coordination of the Mn^II^ ion is slightly distorted octahedral, for which four sites are from two 2,2-bipyridine ligands and the other two are occupied by two N atoms of the two dicyanamido ligands. The distances between the central Mn^II^ ion and the N atoms of the 2,2'-bipyridine ligands are in agreement with the Mn—N bond lengths in other manganese complexes contaning bipyridine ligands (Lopes *et al.*, 2011; Knight *et al.*, 2010; McCann *et al.*, 1998; Lumme & Lindell, 1988; Li *et al.*, 2002). The Mn—N_dicyanamido_ bond lengths are slightly shorter than the Mn—N_bipyridine_ lengths.

### Experimental

The synthesis of the title complex was carried out by reacting Mn(ClO_4_)_2_.6H_2_O, 2,2'-bipyridine and sodium dicyanamide in a molar ratio of 1:2:2 in methanol. After the mixture was stirred for about 15 minutes at room temperature, it was filtrated. The filtrate was left to slowly evaperate in air for about one week to obtain single-crystals suitable for X-ray diffraction with the yield about 50%.

### Refinement

All H atoms bonded to the C atoms were placed using the HFIX command in *SHELXL-97* (Sheldrick, 2008) with C—H distances of 0.93 Å, and were allowed for as riding atoms with *U*_iso_(H) = 1.2*U*_eq_(C). The atom N6 of one the dicyanamido ligands is disordered and was refined with over two sites with equall occupancies.

### Crystal data

**Table:**

[Mn(C_2_N_3_)_2_(C_10_H_8_N_2_)_2_]	*F*(000) = 1020
*M**_r_* = 499.41	*D*_x_ = 1.379 Mg m^−^^3^
Monoclinic, *P*2_1_/*c*	Mo *K*α radiation, λ = 0.71073 Å
Hall symbol: -P 2ybc	Cell parameters from 2314 reflections
*a* = 9.232 (3) Å	θ = 2.7–26.9°
*b* = 16.144 (6) Å	µ = 0.58 mm^−^^1^
*c* = 16.670 (6) Å	*T* = 293 K
β = 104.439 (6)°	Block, yellow
*V* = 2406.0 (15) Å^3^	0.31 × 0.29 × 0.24 mm
*Z* = 4	

### Data collection

**Table:**

Bruker APEXII diffractometer	4220 independent reflections
Radiation source: fine-focus sealed tube	2653 reflections with *I* > 2σ(*I*)
Graphite monochromator	*R*_int_ = 0.040
φ and ω scans	θ_max_ = 25.0°, θ_min_ = 1.8°
Absorption correction: multi-scan (*SADABS*; Sheldrick, 1996)	*h* = −10→10
*T*_min_ = 0.840, *T*_max_ = 0.873	*k* = −19→17
11521 measured reflections	*l* = −9→19

### Refinement

**Table:**

Refinement on *F*^2^	Primary atom site location: structure-invariant direct methods
Least-squares matrix: full	Secondary atom site location: difference Fourier map
*R*[*F*^2^ > 2σ(*F*^2^)] = 0.056	Hydrogen site location: inferred from neighbouring sites
*wR*(*F*^2^) = 0.178	H-atom parameters constrained
*S* = 1.03	*w* = 1/[σ^2^(*F*_o_^2^) + (0.0716*P*)^2^ + 2.6518*P*] where *P* = (*F*_o_^2^ + 2*F*_c_^2^)/3
4220 reflections	(Δ/σ)_max_ = 0.001
325 parameters	Δρ_max_ = 0.91 e Å^−^^3^
0 restraints	Δρ_min_ = −0.79 e Å^−^^3^

### Special details

**Table:**

Geometry. All e.s.d.'s (except the e.s.d. in the dihedral angle between two l.s. planes) are estimated using the full covariance matrix. The cell e.s.d.'s are taken into account individually in the estimation of e.s.d.'s in distances, angles and torsion angles; correlations between e.s.d.'s in cell parameters are only used when they are defined by crystal symmetry. An approximate (isotropic) treatment of cell e.s.d.'s is used for estimating e.s.d.'s involving l.s. planes.
Refinement. Refinement of *F*^2^ against ALL reflections. The weighted *R*-factor *wR* and goodness of fit *S* are based on *F*^2^, conventional *R*-factors *R* are based on *F*, with *F* set to zero for negative *F*^2^. The threshold expression of *F*^2^ > σ(*F*^2^) is used only for calculating *R*-factors(gt) *etc*. and is not relevant to the choice of reflections for refinement. *R*-factors based on *F*^2^ are statistically about twice as large as those based on *F*, and *R*- factors based on ALL data will be even larger.

### Fractional atomic coordinates and isotropic or equivalent isotropic displacement parameters (Å^2^)

**Table:**

	*x*	*y*	*z*	*U*_iso_*/*U*_eq_	Occ. (<1)
Mn1	0.49855 (7)	0.78202 (4)	0.88004 (4)	0.0570 (3)	
N1	0.3997 (4)	0.6864 (2)	0.7812 (2)	0.0592 (9)	
N2	0.6547 (4)	0.7703 (2)	0.7951 (2)	0.0583 (9)	
N3	0.6062 (4)	0.6721 (2)	0.9643 (2)	0.0601 (9)	
N4	0.3479 (4)	0.7467 (2)	0.9633 (2)	0.0590 (9)	
N5	0.6450 (5)	0.8677 (3)	0.9614 (3)	0.0847 (13)	
N6	0.7237 (11)	0.9877 (6)	1.0436 (6)	0.092 (3)	0.50
N6A	0.8534 (16)	0.9247 (8)	1.0702 (7)	0.141 (5)	0.50
N7	0.9807 (6)	1.0470 (3)	1.0971 (4)	0.121 (2)	
N8	0.3514 (5)	0.8769 (3)	0.8116 (3)	0.0906 (13)	
N9	0.2729 (7)	0.9927 (3)	0.7186 (4)	0.1155 (17)	
N10	0.0440 (6)	1.0630 (4)	0.6652 (4)	0.1195 (19)	
C1	0.2698 (5)	0.6462 (3)	0.7764 (3)	0.0737 (13)	
H1	0.2145	0.6602	0.8141	0.088*	
C2	0.2151 (7)	0.5859 (4)	0.7192 (3)	0.0963 (18)	
H2	0.1260	0.5586	0.7187	0.116*	
C3	0.2946 (7)	0.5671 (4)	0.6633 (4)	0.111 (2)	
H3	0.2586	0.5275	0.6227	0.134*	
C4	0.4294 (6)	0.6066 (3)	0.6663 (3)	0.0905 (17)	
H4	0.4857	0.5926	0.6292	0.109*	
C5	0.4786 (5)	0.6672 (3)	0.7258 (2)	0.0587 (11)	
C6	0.6193 (5)	0.7143 (3)	0.7328 (2)	0.0554 (10)	
C7	0.7090 (6)	0.7030 (3)	0.6796 (3)	0.0839 (15)	
H7	0.6829	0.6646	0.6368	0.101*	
C8	0.8388 (7)	0.7491 (4)	0.6898 (4)	0.1022 (19)	
H8	0.9012	0.7414	0.6544	0.123*	
C9	0.8740 (6)	0.8057 (4)	0.7518 (3)	0.0873 (16)	
H9	0.9601	0.8376	0.7591	0.105*	
C10	0.7813 (5)	0.8149 (3)	0.8032 (3)	0.0735 (13)	
H10	0.8064	0.8535	0.8458	0.088*	
C11	0.7362 (5)	0.6361 (3)	0.9617 (3)	0.0779 (14)	
H11	0.7867	0.6552	0.9236	0.094*	
C12	0.7970 (6)	0.5724 (4)	1.0131 (4)	0.0931 (17)	
H12	0.8868	0.5486	1.0092	0.112*	
C13	0.7265 (7)	0.5440 (4)	1.0694 (4)	0.0966 (18)	
H13	0.7674	0.5010	1.1049	0.116*	
C14	0.5926 (6)	0.5798 (3)	1.0735 (3)	0.0856 (15)	
H14	0.5419	0.5612	1.1118	0.103*	
C15	0.5350 (5)	0.6441 (3)	1.0195 (3)	0.0579 (10)	
C16	0.3902 (5)	0.6855 (3)	1.0189 (3)	0.0592 (11)	
C17	0.3024 (6)	0.6625 (4)	1.0715 (3)	0.0934 (17)	
H17	0.3327	0.6197	1.1093	0.112*	
C18	0.1697 (7)	0.7035 (4)	1.0674 (4)	0.117 (2)	
H18	0.1096	0.6886	1.1023	0.141*	
C19	0.1276 (6)	0.7660 (4)	1.0117 (4)	0.0999 (19)	
H19	0.0392	0.7949	1.0086	0.120*	
C20	0.2176 (5)	0.7856 (3)	0.9603 (3)	0.0763 (14)	
H20	0.1874	0.8276	0.9217	0.092*	
C21	0.7146 (8)	0.9118 (4)	1.0047 (3)	0.111 (2)	
C22	0.8884 (9)	1.0045 (4)	1.0761 (4)	0.110 (2)	
C23	0.3004 (7)	0.9280 (4)	0.7692 (4)	0.0995 (19)	
C24	0.1368 (8)	1.0230 (4)	0.6921 (4)	0.0939 (18)	

### Atomic displacement parameters (Å^2^)

**Table:**

	*U*^11^	*U*^22^	*U*^33^	*U*^12^	*U*^13^	*U*^23^
Mn1	0.0651 (4)	0.0580 (4)	0.0505 (4)	−0.0031 (3)	0.0195 (3)	−0.0076 (3)
N1	0.061 (2)	0.064 (2)	0.0530 (19)	−0.0087 (18)	0.0151 (17)	−0.0104 (17)
N2	0.057 (2)	0.068 (2)	0.0509 (19)	−0.0071 (17)	0.0158 (16)	−0.0019 (17)
N3	0.061 (2)	0.064 (2)	0.057 (2)	0.0070 (18)	0.0188 (18)	−0.0044 (18)
N4	0.063 (2)	0.061 (2)	0.057 (2)	0.0064 (18)	0.0209 (18)	−0.0025 (18)
N5	0.116 (4)	0.076 (3)	0.063 (2)	−0.029 (3)	0.025 (2)	−0.012 (2)
N6	0.095 (7)	0.090 (6)	0.102 (7)	−0.020 (6)	0.043 (6)	−0.047 (6)
N6A	0.189 (13)	0.124 (10)	0.090 (7)	−0.084 (10)	−0.006 (8)	0.027 (7)
N7	0.090 (4)	0.086 (4)	0.154 (5)	0.011 (3)	−0.029 (4)	−0.035 (3)
N8	0.108 (4)	0.084 (3)	0.081 (3)	0.023 (3)	0.026 (3)	0.011 (3)
N9	0.123 (4)	0.112 (4)	0.115 (4)	0.027 (4)	0.036 (4)	0.029 (4)
N10	0.102 (4)	0.140 (5)	0.118 (4)	0.010 (4)	0.030 (4)	0.028 (4)
C1	0.070 (3)	0.094 (3)	0.060 (3)	−0.023 (3)	0.022 (2)	−0.018 (3)
C2	0.098 (4)	0.112 (4)	0.084 (4)	−0.050 (4)	0.032 (3)	−0.035 (3)
C3	0.129 (5)	0.116 (5)	0.093 (4)	−0.058 (4)	0.036 (4)	−0.054 (4)
C4	0.104 (4)	0.101 (4)	0.076 (3)	−0.030 (3)	0.040 (3)	−0.040 (3)
C5	0.065 (3)	0.063 (3)	0.048 (2)	−0.005 (2)	0.015 (2)	−0.007 (2)
C6	0.060 (3)	0.060 (2)	0.046 (2)	0.000 (2)	0.0113 (19)	−0.003 (2)
C7	0.089 (4)	0.099 (4)	0.074 (3)	−0.016 (3)	0.039 (3)	−0.022 (3)
C8	0.098 (4)	0.132 (5)	0.096 (4)	−0.023 (4)	0.061 (4)	−0.024 (4)
C9	0.068 (3)	0.115 (4)	0.084 (4)	−0.025 (3)	0.029 (3)	−0.005 (3)
C10	0.070 (3)	0.089 (3)	0.062 (3)	−0.019 (3)	0.017 (2)	−0.008 (3)
C11	0.071 (3)	0.085 (3)	0.082 (3)	0.020 (3)	0.028 (3)	0.003 (3)
C12	0.075 (4)	0.096 (4)	0.103 (4)	0.033 (3)	0.012 (3)	0.002 (4)
C13	0.086 (4)	0.090 (4)	0.110 (5)	0.025 (3)	0.019 (4)	0.032 (4)
C14	0.089 (4)	0.081 (3)	0.085 (3)	0.007 (3)	0.020 (3)	0.020 (3)
C15	0.061 (3)	0.054 (2)	0.057 (2)	0.000 (2)	0.012 (2)	−0.004 (2)
C16	0.063 (3)	0.063 (3)	0.054 (2)	−0.003 (2)	0.017 (2)	−0.006 (2)
C17	0.089 (4)	0.107 (4)	0.097 (4)	0.013 (3)	0.046 (3)	0.031 (3)
C18	0.102 (5)	0.144 (6)	0.132 (5)	0.027 (4)	0.078 (4)	0.039 (5)
C19	0.083 (4)	0.115 (5)	0.116 (5)	0.025 (3)	0.052 (4)	0.005 (4)
C20	0.074 (3)	0.081 (3)	0.080 (3)	0.020 (3)	0.031 (3)	0.001 (3)
C21	0.178 (7)	0.111 (5)	0.053 (3)	−0.087 (5)	0.044 (4)	−0.023 (3)
C22	0.162 (7)	0.082 (4)	0.073 (4)	−0.045 (4)	0.004 (4)	−0.008 (3)
C23	0.114 (5)	0.096 (4)	0.096 (4)	0.032 (4)	0.040 (4)	0.024 (4)
C24	0.104 (5)	0.092 (4)	0.093 (4)	0.027 (4)	0.038 (4)	0.025 (3)

### Geometric parameters (Å, º)

**Table:**

Mn1—N5	2.161 (5)	C3—H3	0.9300
Mn1—N8	2.172 (5)	C4—C5	1.387 (6)
Mn1—N2	2.267 (3)	C4—H4	0.9300
Mn1—N4	2.270 (3)	C5—C6	1.485 (6)
Mn1—N1	2.277 (3)	C6—C7	1.369 (6)
Mn1—N3	2.326 (4)	C7—C8	1.384 (7)
N1—C5	1.346 (5)	C7—H7	0.9300
N1—C1	1.348 (5)	C8—C9	1.357 (8)
N2—C10	1.350 (5)	C8—H8	0.9300
N2—C6	1.354 (5)	C9—C10	1.362 (6)
N3—C15	1.337 (5)	C9—H9	0.9300
N3—C11	1.344 (5)	C10—H10	0.9300
N4—C16	1.343 (5)	C11—C12	1.366 (7)
N4—C20	1.347 (5)	C11—H11	0.9300
N5—C21	1.099 (6)	C12—C13	1.349 (8)
N6—C21	1.379 (10)	C12—H12	0.9300
N6—C22	1.506 (12)	C13—C14	1.381 (7)
N6A—C22	1.326 (12)	C13—H13	0.9300
N6A—C21	1.476 (13)	C14—C15	1.391 (6)
N7—C22	1.082 (7)	C14—H14	0.9300
N8—C23	1.112 (7)	C15—C16	1.492 (6)
N9—C24	1.317 (8)	C16—C17	1.386 (6)
N9—C23	1.328 (8)	C17—C18	1.379 (7)
N10—C24	1.077 (7)	C17—H17	0.9300
C1—C2	1.368 (7)	C18—C19	1.359 (8)
C1—H1	0.9300	C18—H18	0.9300
C2—C3	1.357 (7)	C19—C20	1.371 (7)
C2—H2	0.9300	C19—H19	0.9300
C3—C4	1.387 (7)	C20—H20	0.9300
			
N5—Mn1—N8	95.14 (19)	C6—C7—C8	119.6 (5)
N5—Mn1—N2	92.87 (15)	C6—C7—H7	120.2
N8—Mn1—N2	98.21 (15)	C8—C7—H7	120.2
N5—Mn1—N4	99.18 (14)	C9—C8—C7	119.4 (5)
N8—Mn1—N4	95.75 (16)	C9—C8—H8	120.3
N2—Mn1—N4	160.64 (13)	C7—C8—H8	120.3
N5—Mn1—N1	164.60 (14)	C8—C9—C10	118.9 (5)
N8—Mn1—N1	90.74 (16)	C8—C9—H9	120.5
N2—Mn1—N1	72.18 (12)	C10—C9—H9	120.5
N4—Mn1—N1	94.36 (13)	N2—C10—C9	122.9 (5)
N5—Mn1—N3	90.18 (15)	N2—C10—H10	118.6
N8—Mn1—N3	166.36 (15)	C9—C10—H10	118.6
N2—Mn1—N3	94.05 (12)	N3—C11—C12	122.5 (5)
N4—Mn1—N3	70.96 (12)	N3—C11—H11	118.8
N1—Mn1—N3	87.33 (13)	C12—C11—H11	118.8
C5—N1—C1	118.3 (4)	C13—C12—C11	119.8 (5)
C5—N1—Mn1	117.7 (3)	C13—C12—H12	120.1
C1—N1—Mn1	124.0 (3)	C11—C12—H12	120.1
C10—N2—C6	118.0 (4)	C12—C13—C14	119.1 (5)
C10—N2—Mn1	124.2 (3)	C12—C13—H13	120.5
C6—N2—Mn1	117.7 (3)	C14—C13—H13	120.5
C15—N3—C11	118.2 (4)	C13—C14—C15	118.9 (5)
C15—N3—Mn1	117.6 (3)	C13—C14—H14	120.6
C11—N3—Mn1	124.2 (3)	C15—C14—H14	120.6
C16—N4—C20	118.2 (4)	N3—C15—C14	121.6 (4)
C16—N4—Mn1	119.3 (3)	N3—C15—C16	116.0 (4)
C20—N4—Mn1	122.5 (3)	C14—C15—C16	122.5 (4)
C21—N5—Mn1	176.8 (5)	N4—C16—C17	121.2 (4)
C21—N6—C22	105.5 (8)	N4—C16—C15	116.1 (4)
C22—N6A—C21	110.0 (10)	C17—C16—C15	122.7 (4)
C23—N8—Mn1	165.3 (5)	C18—C17—C16	119.5 (5)
C24—N9—C23	121.4 (6)	C18—C17—H17	120.3
N1—C1—C2	123.5 (4)	C16—C17—H17	120.3
N1—C1—H1	118.2	C19—C18—C17	119.3 (5)
C2—C1—H1	118.2	C19—C18—H18	120.3
C3—C2—C1	117.9 (5)	C17—C18—H18	120.3
C3—C2—H2	121.0	C18—C19—C20	118.9 (5)
C1—C2—H2	121.0	C18—C19—H19	120.6
C2—C3—C4	120.4 (5)	C20—C19—H19	120.6
C2—C3—H3	119.8	N4—C20—C19	123.0 (5)
C4—C3—H3	119.8	N4—C20—H20	118.5
C5—C4—C3	118.7 (5)	C19—C20—H20	118.5
C5—C4—H4	120.6	N5—C21—N6	147.3 (9)
C3—C4—H4	120.6	N5—C21—N6A	146.5 (10)
N1—C5—C4	121.1 (4)	N6—C21—N6A	65.6 (7)
N1—C5—C6	116.2 (3)	N7—C22—N6A	143.0 (11)
C4—C5—C6	122.8 (4)	N7—C22—N6	150.9 (8)
N2—C6—C7	121.1 (4)	N6A—C22—N6	65.9 (7)
N2—C6—C5	116.2 (3)	N8—C23—N9	166.4 (7)
C7—C6—C5	122.7 (4)	N10—C24—N9	162.7 (8)
